# Microbiological Food Safety for Vulnerable People

**DOI:** 10.3390/ijerph120810117

**Published:** 2015-08-21

**Authors:** Barbara M. Lund

**Affiliations:** Institute of Food Research, Norwich Research Park, Norwich, NR4 7UA, UK, E-Mail: barbara.lund@ifr.ac.uk

**Keywords:** vulnerable people, food safety, foodborne pathogens, *Campylobacter*, *Salmonella*, *Listeria*, *Toxoplasma*

## Abstract

Foodborne pathogens are more likely to cause infection and to result in serious consequences in vulnerable people than in healthy adults. People with some increase in susceptibility may form nearly 20% of the population in the UK and the USA. Conditions leading to increased susceptibility are listed. The main factors leading to foodborne disease caused by major pathogens are outlined and examples are given of outbreaks resulting from these factors. Measures to prevent foodborne disease include procedures based on Hazard Analysis Critical Control Point principles and prerequisite programmes and, especially for vulnerable people, the use of lower-risk foods in place of higher-risk products.

## 1. Introduction

A substantial proportion of people show increased susceptibility to foodborne illness compared with healthy adults. This susceptibility can result from chronic or acute illness, medication and/or age. The extent of this increased susceptibility differs according to the cause. In order to protect vulnerable people, who may be in hospitals, care homes or in the community, safeguards are needed in the production and supply of meals, and advice should be publicised to vulnerable people on the avoidance of certain foods.

## 2. Importance and Main Hazards of Foodborne Disease

In the United States it is estimated that each year major pathogens cause over 9 million cases of domestically acquired foodborne illness, over 55,000 hospitalizations and over 1000 deaths [1].

The estimated greatest numbers of cases of foodborne disease in the USA due to known pathogens in 2011 were caused by Norovirus, *Salmonella* spp., *Clostridium perfringens*, *Campylobacter* spp., and *Staphylococcus aureus*, but the greatest numbers of deaths were caused by *Salmonella*, *Toxoplasma gondii*, *Listeria monocytogenes*, Norovirus and *Campylobacter* [1] (Table 1).

**Table 1:** Estimated annual number of domestically acquired foodborne illnesses, hospitalizations and deaths caused by major foodborne pathogens in the USA Data from [1].

Pathogen	Estimated Mean Number of Illnesses	Estimated Mean Number of Hospitalizations	Hospitalization Rate (%)	Estimated Mean Number of Deaths	Case-Fatality Rate
Norovirus	5,461,731	14,663	0.27	149	0.003
*Salmonella* spp. non-typhoidal	1,027,561	19,336	1.88	378	0.037
*Clostridium perfringens*	965,958	438	0.04	26	0.003
*Campylobacter* spp.	845,024	8463	1.00	76	0.009
*Staphylococcus aureus*	241,148	1064	0.44	6	0.002
*E. coli* (STEC) 0157 and non-O157	175,905	2409	1.37	20	0.01
*Shigella* spp.	131,254	1456	1.11	10	0.008
*Yersinia enterocolitica*	97,656	533	0.55	29	0.03
*Toxoplasma gondii*	86,686	4428	5.11	327	0.38
*Listeria monocytogenes*	1591	1455	91.45	255	16.03

Estimates in Canada showed that norovirus, *C. perfringens*, *Campylobacter* spp. and non-typhoidal *Salmonella* accounted for 90% of the pathogen-specific, total cases of foodborne disease [2].

In the UK the estimated number of cases and hospitalizations associated with major foodborne pathogens in 2009 [3] are shown in Table 2.

**Table 2:** Estimated number of food-related cases of disease caused by major foodborne pathogens. UK 2009. Data from [3].

Pathogen	Cases	Hospital Admissions	Hospitalization Rate (%)
*Campylobacter*	280,400	562	0.20
*Clostridium perfringens*	79,570	186	0.23
Norovirus	74,100	332	0.45
*Salmonella* non-typhoidal	33,130	2490	7.52
*E. coli* O157	9886	2233	22.59
*Shigella*	1204	33	2.74
*Listeria monocytogenes*	183	All reported outbreaks were among patients who were hospitalized	
*Toxoplasma gondii* *	>35,000 symptomatic	nr	nr

* From [4], the proportion of cases that are foodborne is not known; nr = not reported.

In the EU in 2013 the majority of reported cases of gastrointestinal disease were associated with *Campylobacter* spp., but the greatest number of deaths were caused by *L. monocytogenes*, with a case-fatality rate of 15.6% [5] (Table 3). The importance of norovirus as the cause of foodborne disease in the EU is illustrated by the report that the largest reported foodborne outbreak in the EU in 2012 was a norovirus outbreak in which 10,950 people were affected [6].

**Table 3:** Reported cases, hospitalization, deaths and case-fatality rates due to main zoonoses in confirmed human cases in the EU, 2013. Data from [5].

Pathogen	Number of Confirmed Human Cases	Hospitalization Rate (%)	Reported Deaths	Case-Fatality Rate (%)
*Campylobacter* spp.	214,779	43.6	56	0.05
*Salmonella* non-typhoidal	82,694	36.0	59	0.14
*Yersinia* spp.	6471	48.4	2	0.05
STEC	6043	37.1	13	0.36
*Listeria monocytogenes*	1763	99.1	191	15.6

The higher hospitalization rates in Table 3 than in Table 1 and Table 2 occur because the cases in Table 3 are reported, confirmed cases, whereas those in Table 1 and Table 2 are estimates, allowing for underreporting of cases which would include many cases that were not hospitalized.

In the Netherlands *T. gondii* and *Campylobacter* spp. were assessed as the foodborne pathogens with the highest disease burden, in terms of Disability-Adjusted Life Years (DALYs), on a population basis, while the burden per case was highest for perinatal listeriosis and congenital toxoplasmosis [7].

According to these reports, major foodborne pathogens in these countries include, *Campylobacter* spp., *Salmonella* spp., Norovirus, *C. perfringens*, shiga-toxin-producing *E. coli* (STEC, *E. coli* O157 and similar bacteria), *L. monocytogenes* and *T. gondii*. The last 2 organisms are particularly important because of their high case-fatality rate. The effects of other foodborne pathogens on vulnerable people have been discussed previously [8,9,10]. For the majority of foodborne pathogens serious disease and deaths are associated particularly with vulnerable populations and people with underlying disease [3].

## 3. Groups of Vulnerable People

People who are particularly susceptible to foodborne disease include the very young, the elderly, and the immune compromised. These people may form nearly 20% of the population in the United States and the United Kingdom [8].Factors that lead to increased susceptibility are shown in Table 4 and discussed by Acheson [11]. Solid organ transplant patients are particularly susceptible to infections [12].

The extent to which vulnerability is increased differs greatly between these groups. An estimate of the relative susceptibility of groups to listeriosis, based on incidence in France has been published [13] (Table 5.). Goulet *et al*. [14] estimated that patients with chronic lymphocytic leukemia were the most vulnerable to listeriosis, with an incidence more than 1000 times greater than that in the population with no risk factors, and listed 14 underlying conditions associated with greater than 100-fold increase in susceptibility.

**Table 4:** Host factors that lead to increased susceptibility to foodborne infection and increased severity of illness.

Primary Immunodeficiency, Caused by a Genetic Defect in Some Component of the Immune System
**Secondary immune deficiencies:**
Immunosuppressive drugs in organ transplantation
Leukaemia
HIV/AIDS
Chemotherapy for cancer
Radiotherapy for cancer
Treatment with corticosteroids
Treatment with inhibitors of tumour necrosis factor e.g. for rheumatoid arthritis, Crohn’s disease
Diabetes, primary and secondary
Pregnancy
Age < 5 years
Age > 65 years
**Other factors:**
Malnutrition , involving protein, calories, vitamins or trace metals
Use of acid-suppressing medication, particularly proton pump inhibitors

**Table 5:** Relative susceptibilities of different subpopulations to listeriosis, calculated using relative susceptibility information from France. Data from [13].

Condition	Relative Susceptibility
Transplant	2584
Cancer—blood	1364
Acquired immunodeficiency syndrome (AIDS)	865
Dialysis	476
Cancer—pulmonary	229
Cancer—gastrointestinal/liver	211
Noncancer liver disease	143
Cancer—bladder and prostate	112
Cancer—gynaecological	66
Diabetes—insulin-dependent	30
Diabetes—noninsulin-dependent	25
Alcoholism	18
Perinatals *	14
Aged > 65 years	7.5
Less than 65 years, no other condition (reference population)	1

* Information from the USA.

Vulnerable groups may show a similar range of susceptibility to other pathogens.

The number of susceptible people will increase with the increase in number of elderly people, many of whom are affected by chronic illnesses, and also with the increasing sophistication of treatments. Many people with increased susceptibility to foodborne disease will be in hospitals, nursing or residential homes. Others will be living in their own homes, and with increasing emphasis on movement of care from hospitals to the community (care in the community) the number of susceptible people in the community is likely to increase.

## 4. Main Foods Associated with Hazards

Based on estimated disease burden, in terms of Quality-Adjusted Life Years (QUALYs), the top 50 pathogen-food combinations in the USA were ranked by Batz *et al.* [15], the top 14 of which are shown in Table 6. An estimate of the main pathogen-food combinations, based on studies in the literature [3], is shown in Table 7.

**Table 6:** Top fourteen pathogen-food combinations in the USA estimated by annual disease burden. Data from [15].

Rank	Pathogen-Food Combination
1	*Campylobacter—*poultry
2	*T. gondii*—pork
3	*L.monocytogenes*—deli meats
4	*S. enterica*—poultry
5	*L. monocytogenes*—dairy
5	Norovirus—complex foods
7	*S. enterica*—complex foods
8	*S. enterica*—produce
8	*T. gondii*—beef
10	*S. enterica*—eggs
11	*L. monocytogenes—*complex foods
12	*S. enterica—*beef
13	*S. enterica—*pork
14	*Norovirus—*produce

**Table 7:** Main foods associated with foodborne disease from food attribution studies in the UK, Canada, Denmark, the Netherlands, USA and the EU. Data from [3].

Pathogen	Foods
*Campylobacter*	Poultry (40%–90%) dairy (10%–40%) red meat (up to 40%)
Norovirus	Seafood up to 40%; produce 20%–40%
*Clostridium perfringens*	Beef & lamb 40%–50%; poultry up to 20%; complex foods up to 20%
*Salmonella*	Eggs 10%–80%; poultry up to 40%; produce up to 20%; poultry, pork, beef and lamb each up to 20%
*E. coli* O157	Beef and lamb 40%–70%; produce 10%–30%
*Listeria*	Unspecified red meat up to 50%; dairy up to 40%; complex foods up to 40%; seafood up to 20%, other meats up to 20%; produce up to 10%

While foods of animal origin are associated with the major proportion of cases of foodborne illness, foods of non-animal origin are also associated with many outbreaks and cases. Studies in the US and the EU have identified top-ranking risk groups of non-animal foods as *E. coli* O157:H7 and leafy greens, *Salmonella enterica* and tomatoes, *S. enterica* and leafy greens, *S. enterica* and melons, and pathogenic *E. coli* and fresh pods, legumes and grains [16,17].

Information from outbreaks and from sporadic cases shows that foods likely to pose a risk of causing foodborne infection, include ready-to-eat foods (*i.e.*, foods intended for consumption without further preparation or heating), that are produced or processed in such a way that does not kill pathogens.

## 5. Main Factors Leading to Foodborne Disease

Main factors leading to foodborne disease include: food from unsafe sources; inadequate cooking; improper holding temperatures, contaminated equipment and cross-contamination and poor personal hygiene.

In the light of these factors, foods that are of particular concern to vulnerable people have been outlined [9]. Examples of outbreaks that have resulted from these factors are shown in Table 8.

Outbreaks of toxoplasmosis are rarely reported, as immune competent people infected usually show mild or no symptoms. A range of outbreaks were described by Smith [18], of 17 outbreaks 13 were associated with consumption of raw or rare meat or raw goat’s milk. Several outbreaks have been associated with drinking water [19].

## 6. Prevention of Foodborne Disease in Vulnerable People

### 6.1. Control of Food Provision

The EU regulation on the hygiene of foodstuffs [20] and Food Hygiene (England) Regulations 2006 and similar regulations in Scotland and Wales, include a requirement that food business operators should put in place, implement and maintain a permanent procedure or procedures based on hazard analysis and critical control point principles (HACCP) [21].This applies to meals supplied in hospitals and institutions as well as other food businesses. In order for a HACCP system to be effective, prerequisite programs must be in place to control factors such as Good Manufacturing Practice, raw material control, production control, pest control, sanitation and maintenance, use of approved suppliers and supplier auditing schemes.

It is particularly important that food for vulnerable groups of people is obtained from reputable suppliers who comply with legal requirements, have in place an appropriate food safety management system based on HACCP principles, and use safe food-handling techniques.

### 6.2. Avoid Food from Unsafe Sources

Avoidance of food from unsafe sources is particularly important in the case of foods that will not receive further processing, such as cooking, before consumption. Such foods include raw or unpasteurized milk, soft or mould-ripened cheese made with unpasteurized milk, unpasteurized fruit and vegetable juices, raw salad vegetables and fruit, shellfish harvested from unclassified areas, raw vegetable sprouts.

**Table 8:** Examples of foodborne outbreaks and contributing factors.

Place, Date	Pathogen	Setting	Cases {Hospitalized} (Deaths)	Food Implicated	Factors Leading to Outbreaks	Reference
Austria, 2006	*C. jejuni, C. coli*	Tertiary care hospital	7 (0) patients, 14 staff (0)	Poultry dishes	Prepared in hospital kitchen with no HACCP system in place	[22]
UK, 2011	*C. jejuni, C. coli*	Wedding party	49 {0} (0)	Chicken liver pâté	Undercooked (cooked to 60 °C)	[23]
USA, 2012	*C. jejuni*	Community	148 {10} (0)	Unpasteurized milk	No inactivation	[24]
USA, 2012	*C. jejuni*	Community	6 {2} (0)	Chicken liver	Undercooked	[25]
USA 2001	*Clostridium perfringens*	Residential care facility for mentally ill	7 {2} (2) Deaths associated with constipation resulting from medication	Thanksgiving meal with turkey	Large amount of food prepared well in advance of serving	[26]
UK, 2005	*Clostridium perfringens*	Buffet lunch at event	54 {nr} (nr)	Chicken curry	Prepared in a domestic kitchen, not registered with local authority, bulk of cooked curry left to cool at ambient temperature for ~10 h.	[27]
USA, 2010	*Clostridium perfringens*	Psychiatric hospital	42 (3) patients, 12 (0) staff. Deaths associated with impaired intestinal motility	Cooked chicken	Cooked ~24 h before serving, not cooled adequately	[28]
USA, 2012–2013	*E. coli* O157	Community	17 {0} (0)	Raw ground beef	Traditional practice, previous outbreaks.	[29]
Germany, 2011	*E. coli* O104:H4	Community	3816 {~800} (54)	Raw, sprouted seeds (fenugreek)	Difficult to disinfect before sprouting.	[30,31]
USA, 2012	*E. coli* O157	Schools, daycare, long-term care facilities	17 {6} (2)	Packaged salad lettuce	Possible contamination during growth in field	[32]
Denmark, 2009	*Listeria*	Meals-on-wheels	7 {7} (2)	Sliced beef with sauces and vegetables, intended for microwave cooking by consumer	Beef had been cooked by the supplier at a lower temperature than usual. Four patient had cancer, one had systemic lupus erythematosus, three were aged >80	[33]
Austria, Germany, Czech Republic 2009–2010	*Listeria*	Community	34 {nr} (8)	‘Quargel’ cheese (Red smear cheese)	Cheese made with pasteurized milk, but contamination probably occurred when cheeses were coated with a culture of *Brevibacterium linens*: subsequent holding at 12 °C–16 °C probably allowed multiplication of listeria	[34,35]
USA, 2010	*Listeria*	Five hospitals	10 {5} (5)	Diced celery, often in sandwiches.	Probably contaminated in the field	[36]
USA 2010–2015	*Listeria*	Hospital	10 {10} (3)	Milkshakes made with ice-cream product	Unsatisfactory hygiene in factory producing ice-cream	[37]
Denmark, 2005	Norovirus	Hospital, nursing homes, meals-on-wheels service, restaurant, company canteen	>1000 {~400} (0)	Imported frozen raspberry pieces,	Contamination during growth/harvesting on small farms	[38]
Germany, 2012	Norovirus	Mainly schools and canteens	~11,000 {38} (0)	Imported frozen strawberries	Possible contamination during growth/harvesting	[39]
Denmark, Finland, Norway, Sweden, 2013	Hepatitis A virus	Community	>106 {nr} (nr)	Imported frozen strawberries	Possible contamination during growth/harvesting	[40,41]
Austria, 2009	Norovirus	Hospital, rehabilitation centre and convalescent home	114 (0) Patients and staff affected	Sliced cold sausage; meat dish with salad; spinach pancake	Contamination by one of five asymptomatic excreters among kitchen staff who prepared food. No HACCP in place	[42]
UK, Norway, France, Sweden, Denmark, 2010	Norovirus	Mainly restaurants	334 cases, 65 clusters {nr} (nr)	Oysters	Probably contaminated in oyster-growing areas and inadequately cooked	[43]
Denmark, 2005	*S.* Typhimurium DT104*	Restaurant	40 {11} (0)	Carpaccio (thinly sliced, raw fillet of beef)	Imported, contaminated beef uncooked	[44]
Netherlands, 2005	*S.* Typhimurium DT104	Community, food from mobile caterers a risk factor	169 {0} (0)	Steak tartare (raw, minced beef)	Imported, contaminated beef uncooked	[45]
Germany, The Netherlands 2011	*S.* Newport	Rehabilitation clinic and Asian restaurants in Germany, hospital in the Netherlands	126 {30} (0)	Mung bean sprouts	Sprouts served raw or undercooked	[46]
Netherlands, 2012	*S.* Thompson	Community	>1149 {>46} (>4)	Smoked salmon	Transport of salmon on reusable, porous dishes on processing lines.	[47]
UK, 2014 (part of multinational outbreak)	*S.* Enteritidis	Hospital canteen, (patients, staff and visitors affected),three restaurants	287 {78} (1)	Eggs from a German producer	Not reported, probably undercooked	[48]

* MDR = multi antibiotic-resistant; nr = not reported.

### 6.3. Ensure Adequate Cooking

Conditions advised for cooking foods (Table 9) are based on a requirement to inactivate vegetative foodborne bacterial pathogens, including *Campylobacter* spp., STEC, *Salmonella* spp. and *L. monocytogenes*, the most heat-resistant of this group.

**Table 9:** Temperatures and times advised for thorough cooking of animal foods: A food thermometer should be used to check the internal temperature reached throughout the food.

Food	Temperature to be Reached in All Parts of the Food	Time at Specified Temperature	Reference
1. Meat, eggs, seafood, minced meats, rolled roasts, large joints of meat, whole poultry, soups, stews poultry	70 °C	-	[49]
2. Burgers(ground, minced meat) poultry livers, and other foods	70 °C	At least 2 min	[50]
	or equivalent temperature/time combination		[51] [52]
3. Raw eggs broken and prepared for immediate service, Fish, meat, except as specified in 4, 5, 6.	63 °C (145 °F) or above	15 s	[53]
4. Mechanically tenderized meat, injected meats, ratites, comminuted fish, meat, game animals commercially raised for food, raw eggs not prepared for immediate service	68 °C (155 °F)	15 s	[53]
	or equivalent temperature/time combination		
	70 °C (158 °F)	<1 s	
	66 °C (150 °F)	1 min	
	63 °C (145 °F)	3 min	
5. Whole meat roasts including beef, corned beef, pork, cured pork roasts such as ham	70 °C (158 °F)	-	[53]
	Or equivalent temperature/time combination, e.g.		
	65 °C (149 °F)	85 s	
6. Poultry, baluts, wild game animals, stuffed fish, stuffed pasta, stuffed poultry stuffed ratites, stuffing containing fish, meat, poultry or ratites	74 °C (165°F) or above	15 s	[53]
7. Raw animal food cooked in a microwave oven	>74 °C (165 °F)	Allow to stand for 2 min	[53]

-, instantaneous.

### 6.4. Ensure Control of Holding Temperature

*Clostridium perfringens* forms spores that are heat-resistant and are not inactivated by cooking. Meals, particularly meat dishes, that are not eaten immediately but are prepared in advance, should be cooled within two hours from 57 °C to 21 °C and within 6 hours from 57 °C to 5 °C [53] and not allowed to remain at temperatures between 12 °C and 50 °C, which can allow the spores to germinate and the vegetative bacteria to multiply to numbers that can cause food-poisoning after consumption of the meal. Meals that are prepared and stored should be reheated to at least 74 °C for 15 sec before consumption [53].

Chilled foods should be maintained in a refrigerator at <5 °C to reduce growth of food-poisoning bacteria and maintain the safety and quality of the food. *Listeria moncytogenes* can grow slowly at temperatures as low as 3 °C–5 °C.

### 6.5. Prevent Cross-Contamination of Foods

Raw foods and ready-to-eat foods should be well-separated. Food-contact surfaces and equipment, including meat slicers, should be cleaned thoroughly. Separate equipment and utensils should be used for each item of raw food and for cooked food.

### 6.6. Maintain Good Personal Hygiene

Guidance in the UK specifies that staff who show symptoms of illness such as diarrhoea and/or vomiting must be excluded from working with or around open food, usually until at least 48 h after symptoms stop naturally [54]. In the case of people with symptoms caused by *S.* Typhi, *S.* Paratyphi, shiga-toxin-producing *E. coli*, and Hepatitis A virus more stringent requirements apply, and clearance by a medical professional is needed before a return to work. In the case of infection with norovirus, because of the ease with which the virus can be spread, it is advised that symptomatic food handlers should be excluded from the entire food business site, and remain away until at least 48 hours after symptoms stop.

Comparable directions are given in the FDA Food Code [53], with similar conditions applied to *Shigella* and STEC infections. More stringent requirements are given for employees working in a food establishment that serves a highly susceptible population than for those not working in such an establishment.

Persons who are asymptomatically infected may excrete small numbers of bacteria or viruses for weeks or more, those who show symptoms may continue to excrete the pathogen after they have recovered from symptoms of infection. Effective hand washing and good hygienic practices are important to prevent risk of infection.

### 6.7. Low—Microbial Diets

For clearly identified, vulnerable groups of people low-microbial diets or neutropenic diets have been advised by many organizations [9].The neutropenic diet prescribed by dieticians in the UK varies greatly [55]. According to Silk *et al*. [56] “evidence for the problem of unsafe food preparation and service for immune compromised and elderly patients is not only widespread, but also underestimated and can be expected to grow”. They proposed that professional organizations should promote, and large healthcare systems should establish, policies to prepare safe food for immune compromised patients and not serve them higher-risk foods, and that such practices could be implemented as the standard for hospital care. With the increase in vulnerable people in residential care and in the community, adherence to HACCP principles and policies for avoidance of higher—risk foods are needed in the supply of meals in these circumstances. It is important that general agreement be reached on recommendations for provision of safe food for vulnerable people and that arrangements should be made to review such recommendations in the light of new information and developments in the microbiological safety of food.

## 7. Conclusions

The number of people in the population with increased susceptibility to foodborne illness is significant and is expected to increase. Many vulnerable people will be in hospitals, nursing or care homes, others will be living in the community. It is particularly important that businesses who supply food to vulnerable people in all these situations should have in place food safety management systems based on Hazard Analysis Critical Control Point principles and safe food-handling techniques. For clearly identified vulnerable people generally agreed advice should be given on avoidance of higher-risk foods and the use of lower-risk alternatives.

## References

[1] Scallan E., Hoekstra R.M., Angulo F.J., Tauxe R.V., Widdowson M.-A., Roy S.H., Jones J.L., Griffin P.M. (2011). Foodborne illness acquired in the United States—Major pathogens. Emerg. Infect. Dis..

[2] Thomas M.K., Murray R., Flockhart I., Pintar K., Pollan F., Fazil A., Nesbitt A., Marshall B. (2013). Estimates of the burden of foodborne illness in Canada for 30 specified pathogens and unspecified agents Circa 2006. Foodborne Pathog. Dis..

[3] Tam C.C., Larose T., O’Brien S.J. Costed Extension to The Second Study of Infectious Intestinal Disease in the Community: Identifying the Proportion of Foodborne Disease in the UK and Attributing Foodborne Disease by Food Commodity Project B18021 (FS231043) 2014. http://www.food.gov.uk.

[4] Advisory Committee on the Microbiological Safety of Food (ACMSF) Ad hoc Group on Vulnerable Groups (2012). Risk Profile in Relation to Toxoplasma in the Food Chain. http://acmsf.food.gov.uk.

[5] European Food Safety Authority (EFSA) (2015). The European Union Summary Report on Trends and Sources of Zoonoses, Zoonotic Agents and Food-Borne Outbreaks in 2013. EFSA J..

[6] European Food Safety Authority (EFSA) (2014). The European Union summary report on trends and sources of zoonoses, zoonotic agents and food-borne outbreaks in 2012. EFSA J..

[7] Havelaar A.H., Haagsma J.A., Mangen M.-J., Kemmeren J.M., Verhoef L.P.B., Vijgen S.M.C., Wilson M., Friesema I.H.M., Kortbeek L.M., van Duynhoven Y.T.H.P. (2012). Disease burden of foodborne pathogens in the Netherlands, 2009. Int. J. Food. Microbiol..

[8] Lund B.M., O’Brien S.J. (2011). The occurrence and prevention of foodborne disease in vulnerable people. Foodborne Pathog. Dis..

[9] Lund B.M. (2014). Microbiological food safety and a low microbial diet to protect vulnerable people. Foodborne Pathog. Dis..

[10] Lund B.M., O’Brien S.J., Motarjemi Y. (2014). Public Health Measures: Food safety in hospitals and other healthcare settings. Encyclopedia of Food Safety.

[11] Acheson D. (2013). Iatrogenic high-risk populations and foodborne disease. Infect. Dis. Clin. N. Am..

[12] Obayashi P.A.C. (2012). Food safety for the solid organ transplant patient: preventing foodborne illness while on chronic immunosuppressive drugs. Nutr. Clin. Pract..

[13] FAO/WHO (2014). Risk Assessment of *Listeria Monocytogenes* in Ready-to-Eat Foods. http://www.fao.org/docrep/010/y5394e/y5394e00.HTM.

[14] Goulet V., Hebert M., Hedberg C., Laurnent E., Vaillant V., de Valk H., Desenclos J.-C. (2012). Incidence of listeriosis and related mortality among groups at risk of acquiring listeriosis. Clin. Infect. Dis..

[15] Batz M.B., Hoffman S., Morris J.G. (2012). Ranking the disease burden of 14 pathogens in food sources in the United States using attribution data from outbreak investigations and expert elicitation. J. Food Prot..

[16] Anderson M., Jaykus L.-E., Beulieu S., Dennis S. (2011). Pathogen-produce pair attribution risk ranking tool to prioritize fresh produce commodity and pathogen combinations for further evaluation (P3ARRT). Food Control.

[17] Da Silva Felίcio M.T., Hald T., Liebana E., Allende A., Hugas M., Nguyen-The C., Skoien Johanesen G., Niskanen T., Uyttendaele M., Mclauchlin J. (2015). Risk ranking of pathogens in ready-to-eat unprocessed foods of non-animal origin (FoNAO) in the EU: Initial evaluation using outbreak data (2007–2011). Int. J. Food Microbiol..

[18] Smith J.L. (1993). Documented outbreaks of toxoplasmosis: Transmission of *Toxoplasma gondii* to humans. J. Food Prot..

[19] Jones J.L., Dubey J.P. (2010). Waterborne toxoplasmosis—Recent developments. Exp. Parasitol..

[20] European Commission (EC) (2004) Regulation (EC) No. 852/2004 of the European Parliament and of the Council of 29 April 2004 on the Hygiene of foodstuffs. Off. J. Eur. Union.

[21] International Life Sciences Institute Europe (ILSI) A Simple Guide to Understanding and Applying the Hazard Analysis Critical Point Concept. http://www.ilsi.org.

[22] Jelovcan S., Schmid D., Lederer I., Hell M., Rehberger K., Arnhold D., Krassnig G., Lassnig H., Romanek G., Pless P. (2008). Cluster of nosocomial campylobacteriosis, Austria 2006. J. Hosp. Infect..

[23] Edwards D.S., Milne L.M., Morrow K., Sheridan P., Verlander N.Q., Mulla R., Richardson J.F., Pender A., Lilley M., Reacher M. (2014). Campylobacteriosis outbreak associated with consumption of undercooked chicken liver pâté in the East of England, September 2011: Identification of a dose-response risk. Epidemiol. Infect..

[24] Longenberger A.H., Palumbo A.J., Chu A.K., Moll M.E., Weltman A., Ostroff S.M. (2013). *Campylobacter jejuni* infections associated with unpasteurized milk—Multiple states, 2012. Clin. Infect. Dis..

[25] Centers for Disease Control and Prevention (CDC) (2013). Multistate outbreak of *Campylobacter jejuni* infections associated with undercooked chicken livers—Northeastern United States, 2012. MMWR Morb. Mortal. Wkly. Rep..

[26] Bos J., Smithee L., McClane B., Distefano R.F., Uzal F., Songer J.G., Mallonee S., Crutcher J.M. (2005). Fatal necrotizing colitis following a foodborne outbreak of enterotoxigenic *Clostridium perfringens* Type A infection. Clin. Infect. Dis..

[27] Holtby I., Tebbutt G.M., Grant K.A., McLauchlin J., Kett J., Pinkney S.A. (2008). *Clostridium perfringens* food poisoning outbreak associated with consumption of chicken curry supplied by a home caterer. Public Health.

[28] Centers for Disease Control and Prevention (CDC) (2012). Fatal foodborne *Clostridium perfringens* illness at a state psychiatric hospital—Louisiana, 2010. MMWR Morb. Mortal. Wkly. Rep..

[29] Centers for Disease Control and Prevention (CDC) (2013). Notes from the field: *Escherichia coli* O157:H7 outbreak associated with seasonal consumption of raw ground beef—Wisconsin, December 2012–January 2013. MMWR Morb. Mortal. Wkly. Rep..

[30] Frank C., Werber D., Cramer J.P., Askar M., Faber M., an der Heiden M., Bernard H., Fruth A., Prager R., Spode A. (2011). Epidemic profile of Shiga-toxin-producing *Escherichia coli* O104:H4 outbreak in Germany. N. Engl. J. Med..

[31] Soon J.M., Seaman P., Baines R.N. (2013). *Escherichia coli* O104:H4 outbreak from sprouted seeds. Int. J. Hygiene Env. Health.

[32] Marder E.P., Garman K.M., Ingram L.A., Dunn J.R. (2014). Multistate outbreak of *Escherichia coli* O157:H7 associated with bagged salad. Foodborne Pathog. Dis..

[33] Smith B., Larsson J.T., Lisby M., Müller L., Madsen S.B., Engberg J., Bangsborg J., Ethelberg S., Kemp M. (2011). Outbreak of listeriosis caused by infected beef meat from a meals-om-wheels delivery in Denmark 2009. Clin. Microbiol. Infect..

[34] Fretz R., Sagel U., Ruppitsch W., Pietzka A.T., Stögere A., Huhulescu S., Heuberger S., Pichler J., Much P., Pfaff G. (2010). Listeriosis outbreak caused by acid curd cheese “Quargel”, Austria and Germany 2009. Euro. Surveill..

[35] Schroder D., Rossmanith P., Glaser K., Wagner M. (2012). Fluctuation in contamination dynamics of *L. monocytogenes* in quargel (acid curd cheese) lots recalled during the multinational listeriosis outbreak 2009/2010. Int .J. Food Microbiol..

[36] Gaul L.K., Farag N.H., Shim T., Kingsley M.A., Silk B.J., Hyytia-Trees E. (2013). Hospital-acquired listeriosis outbreak caused by contaminated celery—Texas, 2010. Clin. Infect. Dis..

[37] CDC Multistate Outbreak of Listeriosis Linked to Blue Bell Creameries Products (Final Update) 2015. http://www.cdc.gov/listeria/outbreaks/ice-cream-03-15/index.html.

[38] Falkenhorst G., Krusell L., Lisby M., Böttiger S., Mølbak K. (2005). Imported frozen raspberries cause a series of norovirus outbreaks in Denmark, 2005. Euro. Surveill..

[39] Mäde D., Trübner K., Neubert E., Höhne M., Johne R. (2013). Detection and typing of norovirus from frozen strawberries involved in a large-scale gastroenteritis outbreak in Germany. Food Environ. Virol..

[40] Gillesberg Lassen S., Soborg B., Midgley S.E., Steens A., Vold L., Stene-Johansen K., Rimhanen-Finne R., Lofdahl M., Sundqvisr L. (2013). Ongoing Multistrain Food-Borne Hepatitis A Outbreak with Frozen Berries as Suspected Vehicle: Four Nordic Countries Affected, October 2012 to April 2003. Euro. Surveill..

[41] Gossner C.M., Seven E. (2014). Three Simultaneous, Food-borne, Multi-country Outbreaks of Hepatitis A Virus Infection Reported in EPIS-FWD in 2013: What does it Mean for the European Union?. Euro. Surveill..

[42] Schmid D., Kuo H.-W., Hell M., Kasper S., Lederer S., Mikula C., Springer B., Allerberger F. (2011). Foodborne gastroenteritis outbreak in an Austrian healthcare facility caused by asymptomatic, norovirus-excreting kitchen staff. J. Hosp. Infect..

[43] Westrell T., Dusch V., Ethelberg S., Harris J., Hjertqvist M., Jourdan-da Silva N., Koller A., Lenglet A., Lisby M., Vold L. (2010). Norovirus Outbreaks Linked to Oyster Consumption in the United Kingdom, Norway, France, Sweden and Denmark, 2010. Euro. Surveill..

[44] Ethelberg S., Sørensen G., Kristensen B., Christensen K., Krusekll L., Hempel-Jørgensen A., Perge A., Nielsen E.M. (2007). Outbreak of multi-resistant *Salmonella* Typhimurium DT104 linked to carpaccio, Denmark, 2005. Epidemiol. Infect..

[45] Kivi M., Hofhuis A., Notermans D.W., Wannet W.J.B., Heck M.E.O.C., van de Giessen A.W., van Duynhoven Y.T.H.P., Stenvers O.F.J., Bosman A., van Pelt W. (2007). A beef-associated outbreak of *Salmonella* Typhimurium DT104 in the Netherlands with implications for national and International policy. Epidemiol. Infect..

[46] Bayer C., Bernard H., Rabsch W., Hiller P., Malomy B., Pfefferkorn B., Frank C., de Jong A., Friesema I., Stark K. (2014). An Outbreak of *Salmonella* Newport Associated with Mung Bean Sprouts in Germany and the Netherlands, October to November 2011. Euro. Surveill..

[47] Friesema I., de Jong A., Hofhuis A., Heck M., van der Kerkhof H., de Jonge R., Hamerycke D., Nage K., van Vilsteren G., van Beek P. (2014). Large outbreak of Salmonella Thompson Related to Smoked Salmon in the Netherlands, August to December 2012. Euro. Surveill..

[48] Inns T., Lane C., Peters T., Dallman T., Chatt C., McFarland N., Crook P., Bishop T., Edge J., Hawker J. (2015). A Multi-Country *Salmonella* Enteritidis Phage Type 14B Outbreak Associated with Eggs from A German Producer: Near “Real-Time” Application of Whole Genome Sequencing and Food Chain Investigations, United Kingdom, May to September 2014. Euro. Surveill..

[49] (2006). World Health Organization (WHO) Five Keys to Safer Food Manual. http://www.who.int.

[50] Advisory Committee on the Microbiological Safety of Food (ACMSF) Report on the Safe Cooking of Burgers. http://www.food.gov.uk/multimedia/pdfs/acmsfburgers0807.pdf.

[51] (2010). Food Standards Agency (FSA) Caterers Warned on Chicken Livers. http://www.food.gov.uk/news/newsarchive/2010/jul/livers.

[52] Advisory Committee on the Microbiological Safety of Food (ACMSF) (2014). Report from the Adhoc Group on Raw, Rare and Low Temperature (RRLT) Cooked Food. http://acmsf.food.gov.uk.

[53] Food and Drug Administration (FDA) Food Code 2013. http://www.fda.gov.

[54] (2009). Food Standards Agency (FSA) Food Handlers: Fitness to Work. http://www.food.gov.

[55] Carr S.E., Halliday V. (2014). Investigating the use of the neutropenic diet: A survey of UK dieticians. J. Hum. Nutr. Diet..

[56] Silk B.J., McCoy M.H., Iwamoto M., Griffin P.M. (2014). Foodborne listeriosis acquired in hospitals. Clin. Infect. Dis..

